# PhIP-driven prostate cancer involves key molecular regulators and immune microenvironment modulation

**DOI:** 10.3389/fimmu.2026.1782240

**Published:** 2026-04-10

**Authors:** Yaoan Wen, Jiangbin Yang, Yuzhe Su, Shaoyuan Chen, Song Zheng, Shaoxing Zhu, Guoqiang Chen, Shuyuan Zhan

**Affiliations:** 1 Department of Urology, Fujian Medical University Union Hospital, Fuzhou, Fujian, China; 2 Department of Urology, The Second Hospital of Longyan, Longyan, Fujian, China

**Keywords:** bioinformatics, machine learning, molecular docking, PhIP, prostate cancer, tumor microenvironment

## Abstract

**Background:**

Exposure to 2-amino-1-methyl-6-phenylimidazo[4,5-b]pyridine (PhIP) may promote the initiation and progression of prostate cancer (PCa); however, its precise molecular mechanisms remain unclear.

**Methods:**

By integrating network toxicology, bioinformatics, and machine learning algorithms, potential molecular targets of PhIP in PCa were identified. Their biological significance was comprehensively evaluated through immune infiltration analysis, single-cell analysis, high-precision molecular docking, molecular dynamics simulations, and experimental validation.

**Results:**

A total of 17 candidate genes associated with PhIP-induced PCa were identified. SHAP analysis identified SLC14A1 as the dominant contributor to model performance. Molecular docking and molecular dynamics simulations suggested that PhIP could form a stable, high-affinity complex with SLC14A1. Experimental validation showed that PhIP exposure induced cytotoxicity and was associated with decreased SLC14A1 expression, while immune infiltration and single-cell analyses further indicated its close association with the tumor immune microenvironment and epithelial localization.

**Conclusion:**

This study offers valuable insights into the potential risks of PhIP exposure in PCa. The key genes and pathways identified may serve as potential biomarkers and therapeutic targets, providing new directions for future research and public health strategies.

## 1 Introduction

Prostate cancer (PCa) represents the primary cause of cancer-related mortality among men globally, ranks as the second most frequently diagnosed malignancy, and constitutes the fifth leading cause of cancer-associated deaths (1). According to the latest United States cancer statistics, prostate cancer is projected to account for approximately 313,780 new cases and 35,770 deaths in 2025 (2). Although the occurrence of prostate cancer is closely related to factors such as age, family history, ethnicity, and genetic variations, its pathogenesis remains unclear. The differences in incidence rates across regions and the increased incidence among men migrating from low-risk to high-risk areas suggest that environmental factors may also play an important role in the development of this disease (3). Several epidemiological studies have demonstrated that dietary factors influence prostate cancer risk; however, findings regarding meat consumption remain inconclusive (3–11). 2-Amino-1-methyl-6-phenylimidazo[4,5-b]pyridine (PhIP), 2-amino-3,8-dimethylimidazo[4,5-f]quinoxaline (MeIQx), 2-amino-3-methylimidazo[4,5-f]quinoline (IQ), and more than 25 other heterocyclic aromatic amines (HAAs) are potent dietary toxins produced during the processing or heat treatment of protein-rich foods (4, 12). Studies have confirmed that HAAs are closely related to a variety of oncological diseases, including colon cancer (13), breast cancer (14), prostate cancer (5), and lymphoid tissue malignancies (15). In 2015, the International Agency for Research on Cancer (IARC) classified red meat as a Group 2A carcinogen (probably carcinogenic to humans) and noted that HAAs from high-temperature cooking may be associated with an increased risk of advanced prostate cancer (6, 7). PhIP is the most abundant in HAAs produced during the high-temperature cooking of red meat (10).

PhIP is a HAA with strong carcinogenic and mutagenic potential (10). Unlike other HAAs, PhIP exhibits preferential susceptibility in the prostate and promotes prostate carcinogenesis in rodents through the formation of DNA adducts and metabolic activation to genotoxic metabolites. Specifically, PhIP first undergoes N-hydroxylation mediated by CYP1A2, generating N-hydroxy-PhIP as a reactive intermediate. Subsequently, NAT1 in prostate tissue catalyzes further O-acetylation, leading to the formation of highly genotoxic electrophilic metabolites that induce DNA adduct formation (4). The main mutations induced by PhIP are the transversions of G: C base pairs to T: A base pairs, which can contribute to prostate cancer in rats. The frequency of gene mutations increases by a factor of 20 (16), and can induce humanized CYP1A mice to develop prostate precancerous lesions that closely resemble those in humans, including inflammation, prostatic intraepithelial neoplasia (PIN), carcinoma *in situ*, and others (17). At the same time, PhIP is also associated with immunosuppression (18). In the human prostate, PhIP is the only dietary meat carcinogen for which DNA adducts have been detected. Additionally, NAT1 in prostate tissue may be the primary enzyme responsible for metabolizing PhIP to its active form that leads to DNA adduct formation (19). All of this evidence supports the potential role of PhIP as a dietary carcinogen in the development of prostate cancer. Notably, Hemelrijck et al. found that PhIP intake was inversely associated with prostate cancer risk in patients with GSTT1/GSTM1 deletions, and the number of deletions was associated with advanced prostate cancer (11). Rohrmann et al. reported that PhIP produced by red meat is positively correlated with high-grade prostate cancer, while its relationship with advanced prostate cancer remains unclear (20). However, despite accumulating experimental and epidemiological evidence, the molecular mechanisms by which PhIP drives prostate carcinogenesis remain incompletely understood. Most existing studies focus on DNA adduct formation or single-gene mutations, whereas a systematic understanding of its multi-target regulatory network and tumor microenvironment interactions is still lacking.

To systematically address the limited understanding of PhIP-driven molecular networks and tumor microenvironment interactions in prostate cancer, a systems-level analytical framework is required. As an emerging interdisciplinary field, network toxicology enables the systematic characterization of multi-component, multi-target toxicity pathways by integrating protein-protein interaction (PPI) networks and network topology analysis (21, 22). By integrating network pharmacology and network biology with multi-omics data from genomics, proteomics, and metabolomics studies, the “toxic characteristics–compound–gene–protein model was constructed and developed (23). The model can systematically describe the toxic mechanisms of PhIP and provide a comprehensive, multidimensional theoretical basis and data support for an in-depth analysis of PhIP’s toxicity characteristics. Molecular docking is a three-dimensional, structure-based computational strategy that predicts and verifies the binding patterns and affinities of ligands to target proteins at the microscopic scale (24). Molecular dynamics simulations quantitatively capture the binding stability of ligand-protein complexes at atomic-level spatial and temporal resolution, providing dynamic structural evidence to elucidate the molecular toxic mechanisms of toxins and thereby revealing novel potential intervention targets (25).

This study aims to elucidate the multiscale oncogenic network of PhIP by integrating bioinformatics techniques, network toxicology, machine learning, single-cell RNA sequencing, molecular docking, molecular dynamics simulations, and experimental validation, thereby providing novel insights into the carcinogenic mechanisms of PhIP.

## 2 Materials and methods

### 2.1 Identification of disease-associated targets

In this study, five transcriptome datasets of PCa were obtained from the NCBI GEO database. Among these, GSE62872 and GSE70768 constituted the discovery cohort, while the remaining three datasets (GSE26126, GSE69223, and GSE200879) formed an independent validation cohort. To minimize batch effects and ensure comparability across cohorts, the “SVA” package was employed to model and correct potential confounders. Subsequently, a parametric empirical Bayes framework was applied to further integrate and adjust for residual batch differences. Finally, the correction results were visualized using principal component analysis (PCA).

### 2.2 Identification of chemical constituents and targets of PhIP

To systematically analyze the chemical characteristics of 2-Amino-1-methyl-6-phenylimidazo[4,5-b]pyridine (PhIP) and its potential targets within the Homo sapiens proteome, this study first retrieved its physicochemical properties, biological parameters, and authoritative 2D structural information (SMILES: CN1C2=C(N=CC(=C2)C3=CC=CC=C3)N=C1N) from the PubChem database (https://pubchem.ncbi.nlm.nih.gov/). Building on this data, a multi-platform integrated strategy was employed for target prediction (26). Specifically, in accordance with the input requirements of each platform, the 2D and 3D chemical structures of PhIP were submitted to three complementary online prediction engines: ChEMBL, SwissTargetPrediction, and PharmMapper. The search scope was restricted to “Homo sapiens” to ensure species specificity of the results. After cross-validation and removal of low-confidence and redundant entries, a total of 2,712 unique candidate proteins were identified. A Venn diagram was then constructed to visually represent the overlap among the predictions from the three algorithms, providing a high-confidence target list for subsequent functional enrichment and pathway analyses.

### 2.3 Analysis of differential gene expression

Differential expression analysis of the transcriptome was performed using the “limma” package. Genes were considered significantly differentially expressed if they met the criteria of an adjusted p < 0.05 and |log_2_FC| ≥ 0.585. A volcano plot was then generated using the “ggplot2” package to provide a comprehensive overview of the distribution of differentially expressed genes. Additionally, a heatmap was created to visualize these significantly differentially expressed genes, facilitating the assessment of their expression patterns and clustering trends across all samples.

### 2.4 Weighted gene co-expression network analysis

This study employed the “WGCNA” package in R to construct a scale-free weighted gene co-expression network. The analysis workflow was as follows: (1) Data preprocessing: The top 5,000 genes exhibiting the highest expression variability were selected to minimize noise from low-abundance transcripts. Hierarchical clustering based on sample expression profiles was conducted to identify and remove outlier samples, ensuring robust network inference; (2) Adjacency matrix construction: The initial adjacency matrix was generated by calculating Pearson correlation coefficients for all gene pairs; (3) Soft-threshold optimization: The scale-free topology fit index was assessed across a predefined gradient of β values. The smallest β value that enabled the network to approximate a scale-free distribution with R² ≥ 0.8 was selected as the optimal soft threshold; (4) Topological overlap and module identification: The adjacency matrix was transformed into a topological overlap matrix (TOM). Gene hierarchical clustering was performed based on TOM dissimilarity, and initial modules were identified using the dynamic tree cut algorithm (minModuleSize = 50, deepSplit = 2). Modules with eigengene correlations exceeding 0.75 were merged (mergeCutHeight = 0.25), resulting in robust co-expression modules; (5) Module–phenotype association: Pearson correlation coefficients between module eigengenes and tumor-normal status were calculated. Modules were categorized as strongly associated (|R| ≥ 0.5, P < 0.05), moderately associated (0.3 ≤ |R| < 0.5, P < 0.05), or weakly/not associated (|R| < 0.3 or P ≥ 0.05); (6) Hub gene identification: Within target modules, hub genes with module membership (kME) > 0.8 were selected based on highest connectivity. These genes were further filtered by differential expression analysis. Ultimately, genes that were both significantly differentially expressed and highly correlated with the module were integrated to construct a candidate gene set for PCa.

### 2.5 Identification of disease targets associated with PhIP

To precisely identify the key effector molecules of PhIP in prostate cancer (PhIP-PCa), this study utilized three gene sets: differentially expressed genes (DEGs), WGCNA hub genes, and predicted target genes of PhIP. A three-way intersection was performed in the R environment, and the overlapping genes were defined as the “PhIP-PCa target genes.” The overlap was visualized using a Venn diagram generated with the “VennDiagram” package. Subsequently, the core target gene set was imported into the STRING database 11.5 (confidence score ≥ 0.4, species restricted to “Homo sapiens”) to construct a protein-protein interaction (PPI) network. Topological analysis and visualization were then conducted using Cytoscape 3.10 to elucidate the interactions among the target genes and their expression characteristics in tumors.

### 2.6 Functional enrichment analysis

Referring to previous studies (27), we performed KEGG and Reactome pathway enrichment analyses, as well as Gene Ontology (GO) functional annotation, using the clusterProfiler package (v4.14.6). The C2 collection was extracted from the MSigDB database (**Supplementary Table 1**), and cancer-related keywords were matched via regular expressions to screen 263 and 241 cancer-associated pathways from the original 844 KEGG pathways and 1,787 Reactome pathways, respectively. After merging, a total of 504 pathways were retained for subsequent analyses. Using the previously identified PhIP-PCa intersecting genes as input, we set the minimum gene set size to 5 and the maximum to 500, applying the Benjamini-Hochberg method for multiple testing correction, with the significance threshold set at an FDR-adjusted p-value < 0.05. For GO functional annotation, gene symbols were converted to Entrez IDs using the org.Hs.eg.db database, and enrichment analyses were conducted separately for the three ontology categories: biological process (BP), molecular function (MF), and cellular component (CC). The Fold Enrichment value (the ratio of the proportion of enriched genes to the proportion of background genes) was calculated to evaluate enrichment intensity, and the top five significantly enriched pathways and entries in each GO category were selected for visualization.

### 2.7 Core gene identification utilizing machine learning techniques

To systematically identify PhIP-related diagnostic biomarkers for prostate cancer (PCa), we used the candidate genes derived from the intersection of differentially expressed genes and WGCNA module genes (described above) as the predefined feature set for subsequent machine learning-based prioritization. The detailed procedures are as follows: (1) Using the transcriptomic expression profiles of the discovery cohort, we applied 12 base algorithms—Elastic Net (Enet), Least Absolute Shrinkage and Selection Operator (Lasso), Ridge regression, Stepwise Generalized Linear Model (GLM), Support Vector Machine (SVM), Linear Discriminant Analysis (LDA), GLM Boosting, Partial Least Squares-GLM (PLS-GLM), Random Forest, Gradient Boosting Machine (GBM), eXtreme Gradient Boosting (XGBoost), and Naive Bayes—to screen predictive features from the genome-wide expression profiles. (2) Based on the preselected feature subset, the same 12 machine learning algorithms were used to establish the final diagnostic models. The diagnostic efficacy of different feature–algorithm combinations was systematically evaluated through 127 combinatorial patterns. (3) Regularized models (Enet, Lasso, Ridge) and gradient boosting models (GBM, XGBoost, GLMBoost) optimized key hyperparameters (e.g., regularization strength λ, number of iterations) via 5- to 10-fold cross-validation; stepwise regression and tree-based models were constructed based on information criteria or preset parameters. The reproducibility of results was ensured by setting a random seed (set.seed (123)). (4) The area under the receiver operating characteristic curve (AUC) was used as the core performance metric to rigorously evaluate each model. (5) The average AUC value of each model across cohorts was calculated for efficacy ranking, and complexity assessment was conducted in combination with the number of genes selected by the model. The final screening strategy prioritized models with top-ranked average AUC and a moderate number of genes to balance predictive accuracy and clinical interpretability. The ComplexHeatmap package was used to generate AUC heatmaps, visually demonstrating the diagnostic stability of each model across different cohorts.

### 2.8 Interpretation of the model

Given the inherent “black box” nature of machine learning models, this study introduces the Shapley Additive Explanations (SHAP) algorithm to quantify the marginal contribution of each feature to the predicted output. This method assigns a SHAP value to each gene trait based on Shapley value theory, enabling an interpretable and quantifiable assessment of its positive or negative influence on the model’s decision-making process.

### 2.9 Core gene identification

To elucidate the potential functional roles of core genes in prostate cancer, this study integrated transcriptomic data from The Cancer Genome Atlas (TCGA) and performed a two-step analytical approach focusing on SHAP-ranked core genes. Initially, differential expression analysis was conducted to compare candidate gene expression levels between tumor and normal tissue samples within the TCGA-PRAD dataset. Appropriate statistical methods, such as the t-test or Wilcoxon rank-sum test, were employed to identify core genes exhibiting significant dysregulation in tumor tissues relative to normal controls.

Subsequently, the subset of core genes identified through differential expression analysis underwent survival analysis. This analysis utilized clinical data from the TCGA-PRAD cohort, with progression-free survival (PFS) defined based on biochemical recurrence (BCR) events as annotated in the curated TCGA clinical follow-up records. Survival time was calculated from the date of initial diagnosis to the occurrence of the first documented BCR event, with patients lacking recurrence censored at their last follow-up. For each candidate gene, patients were dichotomized into high- and low-expression groups according to the median expression value within the cohort. Kaplan–Meier survival curves were generated using the “survival” and “survminer” packages in R, and group differences were evaluated via the two-sided log-rank test. To control for multiple hypothesis testing, the Benjamini–Hochberg procedure was applied to adjust the false discovery rate (FDR). Unless otherwise indicated, statistical significance was defined as a two-sided p-value less than 0.05.

### 2.10 Analysis of molecular docking interactions

We utilized the CB-Dock2 server (28) to perform blind molecular docking for a systematic evaluation of the binding characteristics between PhIP and core target proteins. The procedure strictly followed the established protocol: (1) Protein structures were obtained from the RCSB PDB database with a resolution of ≤ 2.5 Å, and the ligand PhIP (PubChem CID: 135700) was uploaded in SDF format; (2) The platform automatically performed protein side-chain repair, hydrogen addition, water removal, ligand 3D construction, charge assignment, and format conversion; (3) The CurPocket algorithm identified potential cavities, and FP2 fingerprints were used to screen templates in the BioLip database with ligand similarity ≥ 0.4. When templates were available, AutoDock Vina structure docking and FitDock template docking were simultaneously initiated, using a grid box edge length of 25 Å and sampling 20 conformations. The results were merged based on Vina scores within the same cavity, retaining the optimal conformation; (4) The conformation with a cluster size > 20% and the lowest ΔG_bind was selected as the final complex. Key interactions, including hydrogen bonds, hydrophobic contacts, and π-π interactions, were analyzed using NGL Viewer and the PLIP module, with residue contact thresholds following CASP standards (≤ van der Waals radius + 0.5 Å). Finally, the resulting protein-ligand complexes were imported into LigPlot^+^ v2.3.1 to generate and output 2D interaction diagrams, facilitating the analysis of key binding site residues and interaction types.

### 2.11 Analysis of molecular dynamics simulations

Molecular dynamics (MD) simulations were performed with the GROMACS 2020 software suite. Topologies and force-field parameters were generated automatically by combining the internal pdb2gmx utility with the AutoFF web server. The receptor was described using the AMBER14SB force field, while the ligand was treated with the GAFF2 force field. The complex was solvated in a cubic TIP3P water box extending at least 1.0 nm from any solute atom (29), and the net charge was neutralized by randomly replacing selected water molecules with appropriate counter-ions using the gmx genion tool. Long-range electrostatic interactions were handled by the Particle Mesh Ewald (PME) algorithm with a real-space cutoff of 1.0 nm; van der Waals interactions were truncated at the same distance. All bonds involving hydrogen atoms were constrained with the SHAKE algorithm, allowing an integration time step of 1 fs in conjunction with the leap-frog Verlet integrator.

Prior to productive sampling, the system underwent a multi-stage energy minimization protocol: 3,000 steps of steepest descent followed by 2,000 steps of conjugate-gradient refinement. Optimization was performed hierarchically—first on water molecules, then on counter-ions, and finally on the entire assembly—while restraining the non-optimized components with harmonic position restraints (1,000 kJ mol^-1^ nm^-2^). Subsequently, 100 ns of NPT simulation were conducted at 310 K and 1 bar; temperature and pressure were maintained using the Nosé–Hoover thermostat (τ_T = 1 ps) and the Parrinello–Rahman barostat (τ_P = 2 ps), respectively. Trajectories were analyzed with GROMACS built-in utilities: gmx rmsd, gmx rmsf, gmx hbond, gmx gyrate, and gmx sasa were used to compute the root-mean-square deviation (RMSD), root-mean-square fluctuation (RMSF), number of hydrogen bonds, radius of gyration (Rg), and solvent-accessible surface area (SASA). The binding free energy of the complex was estimated with the g_mmpbsa plugin employing the MM-PBSA approach, ensuring thermodynamic consistency throughout the analysis.

### 2.12 Immunohistochemical analysis using the Human Protein Atlas

Immunohistochemical (IHC) data were obtained from the Human Protein Atlas (HPA; https://www.proteinatlas.org/). Publicly accessible IHC images, along with their associated annotation data generated using HPA-validated antibodies, were compiled. Protein expression profiles were analyzed in both normal tissues and tumor specimens. Staining attributes, such as staining intensity, the proportion of positively stained cells, and subcellular localization, were assessed in accordance with the standardized evaluation criteria established by the HPA database. The collected IHC images were subsequently employed for qualitative comparative analysis of protein expression patterns across various tissue types.

### 2.13 Cell culture

The human prostatic epithelial cell line RWPE-1 and the human prostate cancer cell line PC-3 were obtained from the American Type Culture Collection (ATCC). All cells were maintained at 37 °C in a humidified incubator with 5% CO_2_. PC-3 cells were cultured in RPMI 1640 medium supplemented with 10% fetal bovine serum (FBS) (Gibco, Grand Island, NY, USA). RWPE-1 cells were maintained in keratinocyte growth medium supplemented with recombinant human epidermal growth factor (5 ng/mL) and bovine pituitary extract (0.05 mg/mL).

### 2.14 Cell viability assay

RWPE-1 cells were plated at a density of 5,000 cells per well in 96-well plates and allowed to incubate overnight. Subsequently, the cells were exposed to varying concentrations of PhIP (Sangon Biotech, Shanghai, China) ranging from 0 to 100 μM for a duration of 48 hours. Cell viability was evaluated utilizing the Cell Counting Kit-8 (CCK-8, AbMole BioScience, Shanghai, China) in accordance with the manufacturer’s protocol, with absorbance readings taken at 450 nm using a microplate reader. All assays were conducted in triplicate. The final concentration of DMSO was maintained below 0.1% across all treatment groups.

### 2.15 Western blotting

PC-3 cells were exposed to PhIP (100 μM) or a vehicle control for the specified time intervals. Total cellular proteins were extracted utilizing RIPA lysis buffer(Beyotime Biotechnology, Shanghai, China) supplemented with protease inhibitors. Protein concentrations were quantified using a BCA protein assay kit. Equivalent amounts of protein were resolved by SDS–PAGE and subsequently transferred onto polyvinylidene difluoride (PVDF) membranes. The membranes were blocked with 5% non-fat milk in Tris-buffered saline containing 0.1% Tween-20 (TBST, Beyotime Biotechnology, Shanghai, China) for one hour at ambient temperature, followed by overnight incubation at 4 °C with primary antibodies targeting SLC14A1(1:500, Thermo Fisher Scientific, Massachusetts, USA) and β-actin(1:1000, Bioworld Technology, MN, USA). After washing with TBST, the membranes were incubated with appropriate horseradish peroxidase–conjugated secondary antibodies for one hour at room temperature.

### 2.16 Real-time quantitative PCR

Total RNA was extracted and quantified using TRIzol reagent (Vazyme Biotech Co., Ltd., Nanjing, China) according to the manufacturer’s instructions. One microgram of RNA was reverse transcribed into cDNA using the HiScript^®^ III First Strand cDNA Synthesis Kit (Vazyme Biotech Co., Ltd., Nanjing, China). Quantitative PCR was performed using ChamQ Universal SYBR^®^ qPCR Master Mix (Vazyme Biotech Co., Ltd., Nanjing, China) on a QuantStudio™ Dx Real-Time PCR System (Applied Biosystems, MA, USA). The reaction program was as follows: initial denaturation at 95 °C for 30 seconds, followed by 35 cycles of denaturation at 95 °C for 5 seconds and annealing/extension at 60 °C for 30 seconds. GAPDH was used as the internal control, and relative gene expression levels were calculated using the 2^(-ΔΔCt) method.

### 2.17 Quantification of immune cell infiltration

The CIBERSORT algorithm was employed to quantitatively characterize the landscape of immune cell infiltration within the tumor microenvironment (TME) of individual PCa patients. Specifically, the algorithm was applied to estimate the relative proportions of 22 tumor-infiltrating immune cell subsets using a predefined leukocyte gene signature matrix (LM22). The analysis was performed using the “CIBERSORT” R package with 1,000 permutations, and only samples with a P-value < 0.05 were considered reliable for downstream analysis. To enhance the robustness of the analysis and reduce algorithm-specific bias, two additional deconvolution methods, EPIC and MCPcounter, were also applied to the same gene expression dataset. EPIC was used to estimate the relative fractions of immune and stromal cell populations, while MCPcounter provided marker-based abundance scores for major immune cell lineages. The consistency across methods was evaluated by calculating Spearman correlation coefficients for shared immune cell categories.

### 2.18 Single cell analysis

The single-cell transcriptome dataset GSE193337 was obtained from the NCBI-GEO database and processed using the standard Seurat 4.3.0 workflow. After importing the raw matrix with Read10X, low-quality cells were filtered based on the following criteria: nFeature between 200 and 8000, nCount greater than 200, with the top 3% highest-count cells excluded, percent.mt below 20% and percent.hb below 1%. Data normalization was performed using LogNormalize, selecting the top 2000 highly variable genes. PCA dimensionality reduction was conducted using 20 principal components (npcs = 20), followed by batch correction with Harmony. Uniform Manifold Approximation and Projection (UMAP) was used for visualization. Manual cell type annotation was performed based on established gene lists from prior studies (30). To validate these manual annotations, seven major human immune reference datasets were integrated, and SingleR was utilized for automated cell type annotation and mapping. This analysis pipeline enabled the identification of specific cell populations expressing key biomarkers.

To investigate the role of SLC14A1 in cell-cell communication within the TME, epithelial cell subsets were isolated from tumor samples and categorized into two groups based on the presence or absence of SLC14A1 expression. CellChat (v2.1.2) was employed to infer intercellular communication using the Secreted Signaling database. Communication filtering was applied with a minimum cell count threshold of 10, and communication probabilities were calculated and aggregated at the signaling pathway level. Differences in the number and strength of communications between SLC14A1^+^ and SLC14A1^-^ epithelial cells and other cell subsets were visualized using chord diagrams, bubble plots, and heatmaps.

## 3 Results

### 3.1 Structural characterization of PhIP and prediction of its putative targets

**Figure 1:**
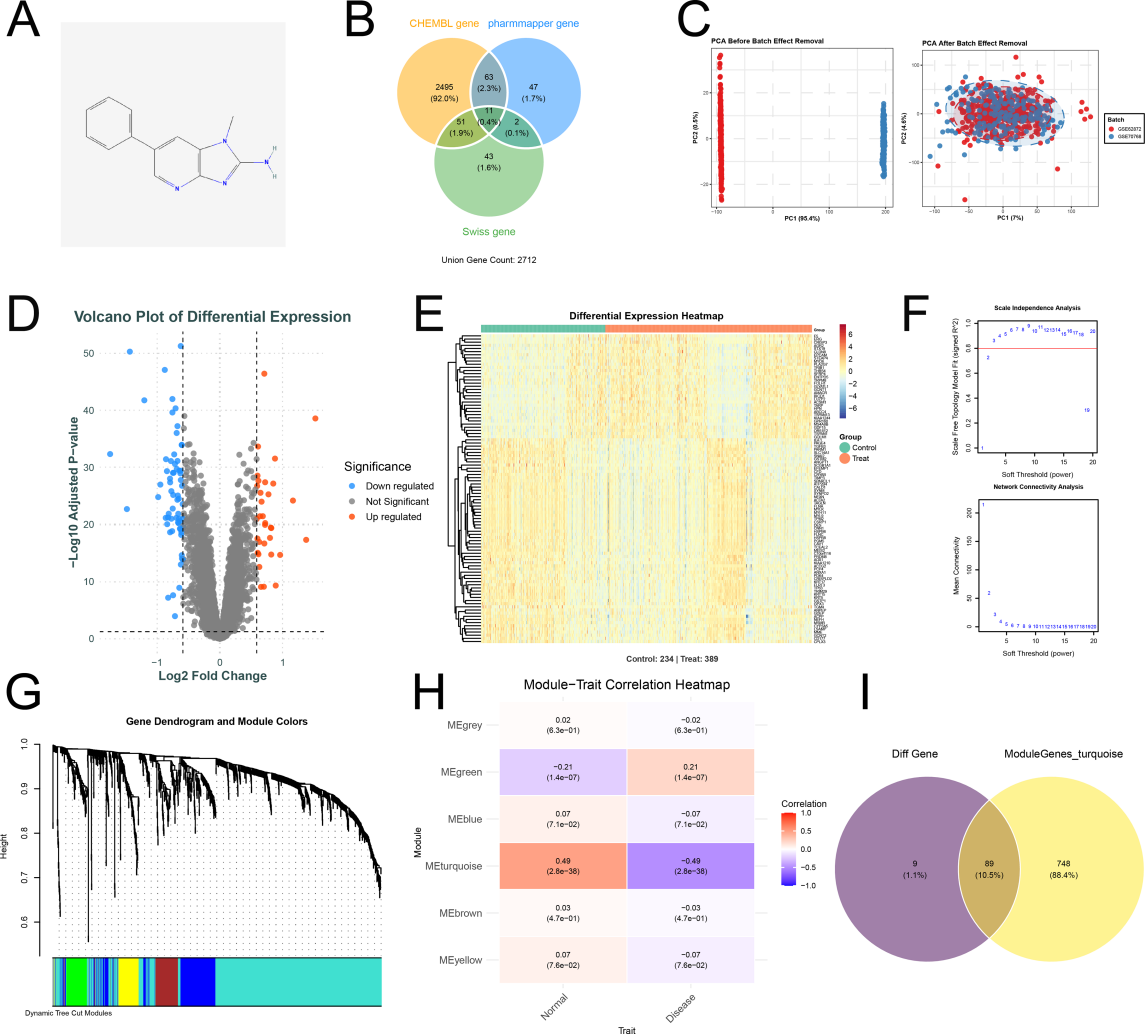
Multi-level integrative analysis of PhIP targets and prostate cancer targets. **(A)** Chemical structure of 2-amino-1-methyl-6-phenylimidazo[4,5-b]pyridine (PhIP). **(B)** Venn diagram showing the overlap of potential PhIP targets identified from three major databases—ChEMBL, SwissTargetPrediction, and PharmMapper—resulting in a total of 2,712 consensus target proteins. **(C)** Comparison of PCA plots before and after batch correction for datasets GSE62872 and GSE70768, demonstrating the effective reduction of batch effects. **(D)** Volcano plot of differentially expressed genes (DEGs) between prostate cancer (PCa) and adjacent normal tissues. Red dots represent significantly upregulated genes in PCa; blue dots represent significantly downregulated genes (adjusted p < 0.05, |log_2_ FC| ≥ 0.585); gray dots indicate genes without significant differential expression. **(E)** Heatmap of the DEGs across all samples, with red indicating high expression and blue indicating low expression. **(F)** WGCNA scale-free topology fit analysis identified a soft-thresholding power β = 3 as the minimum threshold required to construct an approximate scale-free network (R² ≈ 0.85). **(G)** Gene clustering dendrogram based on topological overlap, with color bars below indicating distinct co-expression modules. **(H)** Heatmap of module-trait relationships displaying correlation coefficients and corresponding p-values between each module and clinical traits. **(I)** Venn diagram integrating DEGs (purple), core genes from WGCNA modules (yellow), and their intersection (ochre), ultimately identifying 89 candidate target genes highly associated with PCa.

We retrieved the molecular structure of PhIP from the PubChem database (**Figure 1A**) and predicted its target genes through the three complementary databases: ChEMBL, PharmMapper, and SwissTargetPrediction. By merging the results and removing duplicates, we identified a total of 2,712 potential biological targets for subsequent analysis (**Figure 1B**). To minimize batch effects, the GEO datasets GSE62872 and GSE70768 were first integrated, and the resulting gene expression matrix was systematically normalized. PCA confirmed that the normalized expression profiles showed significant convergence in spatial distribution (**Figure 1C**). Subsequently, a total of 98 genes exhibiting significant differential expression in prostate cancer were identified. Differences in gene expression were illustrated using volcano plots and clustered heat maps (**Figures 1D, E**). In weighted gene correlation network analysis (WGCNA), we first identified the soft-thresholding power β. A systematic evaluation across the range of 1 to 20 indicated that the network topology approximated a scale-free distribution when β = 3 (**Figures 1F**, scale-free R² ≥ 0.8). Using this parameter, a topological overlap matrix (TOM) was constructed, followed by hierarchical clustering to identify five color-coded co-expression modules (**Figure 1G**). Module-trait association analysis revealed that several modules were significantly correlated with PCa. Among them, the MEturquoise module—the largest identified module—exhibited a negative correlation with the “PCa tumor” trait (r = -0.49, p = 2.8 × 10^-38^, **Figure 1H**). Further integration of differentially expressed genes with WGCNA module genes resulted in the identification of 89 non-redundant candidate genes closely associated with the occurrence and progression of PCa (**Figure 1I**).

### 3.2 PhIP-associated disease targets in PCa

**Figure 2:**
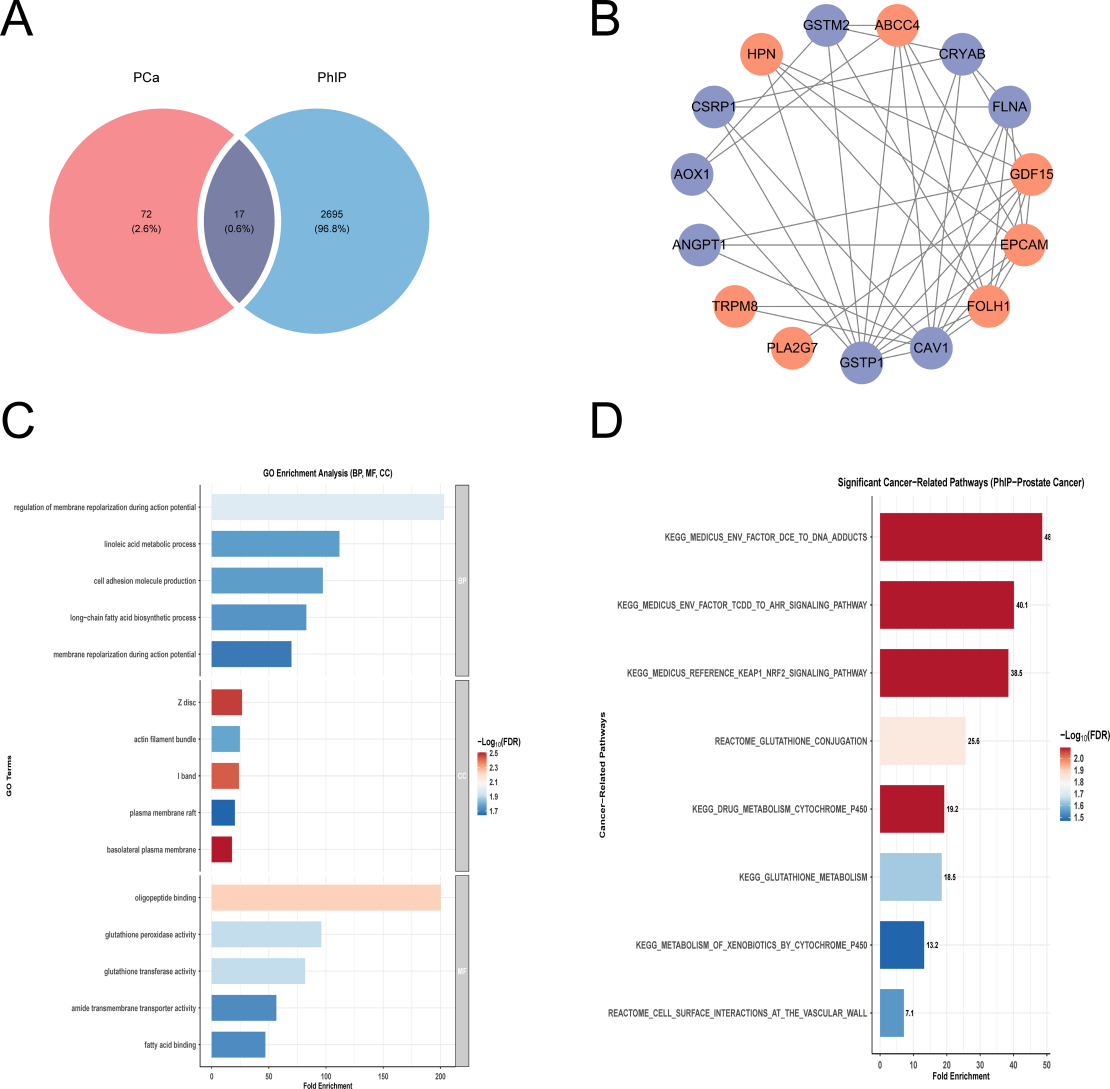
Identification and functional annotation of overlapping targets between PhIP and prostate cancer. **(A)** Venn diagram illustrating the overlap between PhIP consensus targets (blue) and prostate cancer (PCa) highly related targets (red), highlighting a total of 17 overlapping genes (purple). **(B)** Protein-protein interaction (PPI) network of overlapping genes: red nodes represent upregulated genes in PCa, blue nodes represent downregulated genes, and edges indicate predicted protein-protein interactions derived from the STRING database. **(C)** GO functional enrichment analysis of overlapping genes, including biological process, cellular component, and molecular function categories. **(D)** KEGG and Reactome pathway enrichment analysis.

By systematically integrating PhIP target proteins with prostate cancer-related gene sets, we identified a total of 17 potential key nodes that are hypothesized to play a central role in PhIP-induced prostate cancer (**Figure 2A**). The PPI network depicted the relationships among these critical nodes and their corresponding expression levels in prostate cancer cells, with red indicating upregulation and blue indicating downregulation (**Figure 2B**). To further investigate the functional characteristics of 17 candidate targets, we performed Gene Ontology (GO) functional annotation and KEGG/Reactome pathway enrichment analyses using the clusterProfiler package. GO functional annotation (**Figure 2C**) revealed that, at the biological process (BP) level, the target genes were significantly enriched in terms such as regulation of membrane repolarization during action potential, linoleic acid metabolic process, cell adhesion molecule production, and long-chain fatty acid biosynthetic process. These findings suggest that PhIP may contribute to prostate carcinogenesis by modulating membrane potential stability and lipid metabolism. At the cellular component (CC) level, enriched terms were primarily associated with the Z disk, actin filament bundle, I band, plasma membrane raft, and basolateral plasma membrane, indicating that these genes may play crucial roles in cytoskeleton organization and membrane signal transduction. At the molecular function (MF) level, significantly enriched functions included oligopeptide binding, glutathione peroxidase activity, glutathione transferase activity, amide transmembrane transporter activity, and fatty acid binding, highlighting the central roles of the antioxidant defense system and xenobiotic transport and metabolism. Pathway enrichment analysis further demonstrated (**Figure 2D**) that the candidate gene set was significantly enriched in xenobiotic metabolism and detoxification-related pathways, including DNA adduct formation (Fold Enrichment = 48.0), aryl hydrocarbon receptor (AHR) signaling pathway (40.1), KEAP1–NRF2 signaling pathway (38.5), glutathione binding (25.6), cytochrome P450 drug metabolism (19.2), glutathione metabolism (18.5), and cytochrome P450–mediated xenobiotic metabolism (13.2). These findings are highly consistent with the metabolic activation mechanism of PhIP as a heterocyclic amine carcinogen.

### 3.3 Core genes in PhIP-induced PCa

**Figure 3:**
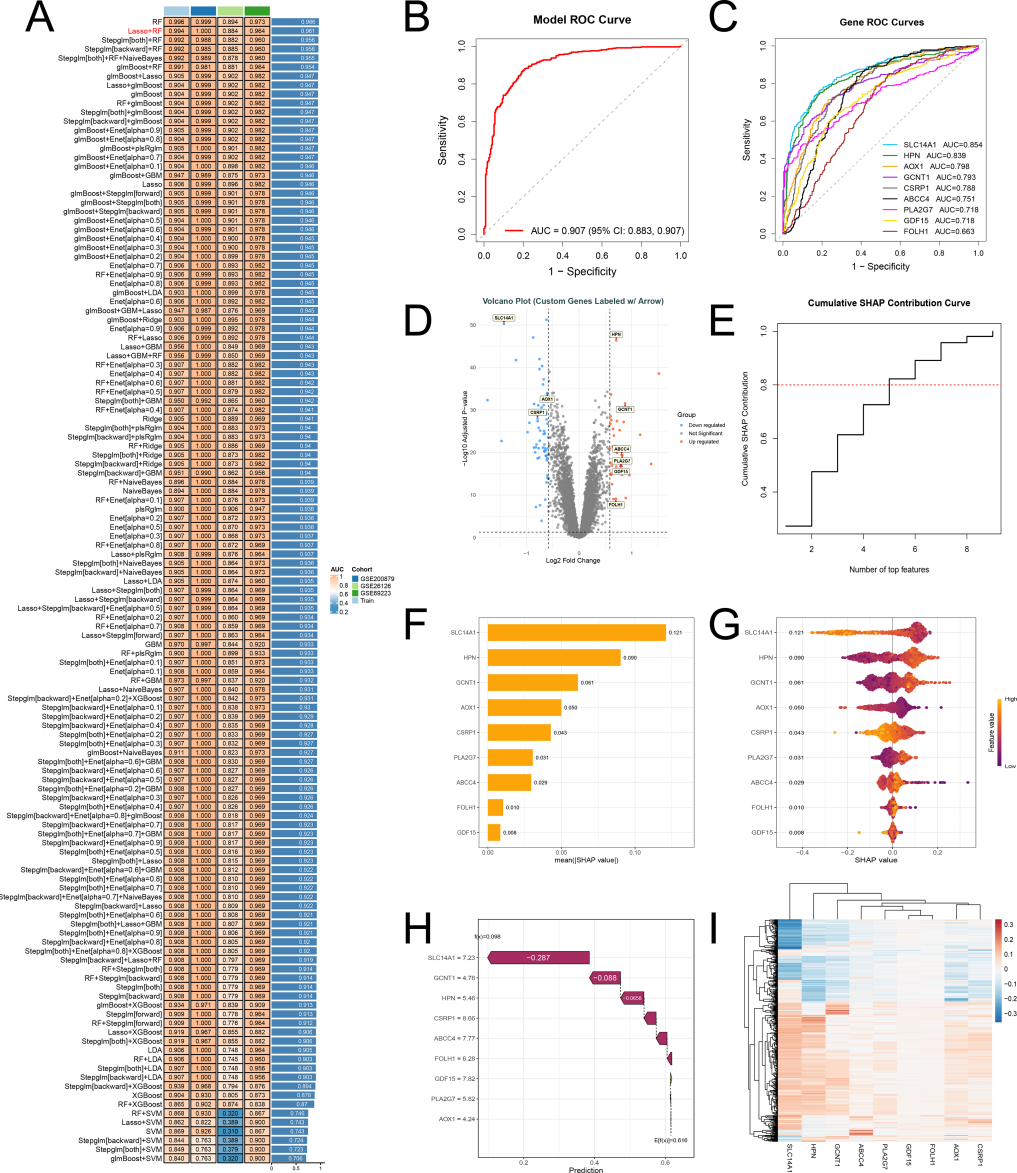
Screening and interpretability analysis of prostate cancer core genes using 127 machine learning models. **(A)** Heatmap comparing the performance of multi-cohort models. Algorithm names are listed on the left, with corresponding AUC values on the right. The color gradient visually represents differences in predictive performance. Lasso+RF was selected as the final algorithm. **(B)** Overall ROC curve of the Lasso+RF ensemble model, showing an AUC of 0.907, which indicates excellent discriminative ability. **(C)** Individual ROC curves for nine core genes: SLC14A1 exhibited the best single-gene performance (AUC = 0.854), while the other genes also demonstrated notable discriminative power. **(D)** Volcano plot of differential gene expression in PCa, highlighting the nine core genes based on significance criteria (adjusted p < 0.05 and |log_2_FC| ≥ 0.585). **(E)** Cumulative SHAP contribution curve: illustrating the increasing cumulative explanatory power of core genes on the model output as the number of genes increases. **(F)** Bar chart of core gene SHAP contributions, ranked in descending order by absolute mean values, quantifying the importance of each gene. **(G)** SHAP beeswarm plot: The x-axis represents SHAP values (indicating direction and magnitude of influence), the y-axis lists genes. Colors range from purple (low expression) to yellow (high expression), illustrating the relationship between gene expression levels and their contributions. **(H)** SHAP waterfall plot for a representative sample: analyzing the dynamic contribution of each gene to a single prediction, from the baseline value to the final output. **(I)** Sample-by-gene SHAP value clustering heatmap: color intensity reflects SHAP magnitude, revealing heterogeneous patterns of core gene contributions across different samples.

Using the 89 non-redundant candidate genes as the predefined input feature matrix, we systematically developed 127 combinatorial patterns to identify the core driver genes of PhIP-associated prostate cancer. The results show that although the RF model (11 genes) ranked first in the heatmap, the Lasso+RF ensemble strategy, which ranked second, reduced the feature set to 9 genes with an AUC loss of less than 0.005 and demonstrated excellent discriminative performance in both the training and independent validation sets (**Figure 3A**). Consequently, the model identified 9 core genes: SLC14A1, HPN, AOX1, GCNT1, CSRP1, ABCC4, PLA2G7, GDF15, and FOLH1. ROC curve analysis further validated the robust diagnostic value of these 9 genes for PhIP-related prostate cancer (**Figures 3B, C**), and the volcano plot offers an intuitive visualization of the positions of these genes within the set of differentially expressed genes (**Figure 3D**).

The cumulative SHAP contribution curve shows that the top 5 features account for over 80% of the model’s SHAP importance, indicating that the model heavily relies on a few key features (**Figure 3E**). Therefore, we focused on these top 5 genes in subsequent analyses. Using SHAP interpretability analysis, we calculated the contribution of each gene to the predicted outcome and visualized the results (**Figure 3F**). Among these, SLC14A1 was the most influential predictor (SHAP = 0.121). Samples with high SLC14A1 expression suppressed the model output, indicating a lower likelihood of being predicted as prostate cancer. When expression was low, this effect was diminished or slightly positive. AOX1 exhibits effects similar to those of SLC14A1, although their impact on the model is significantly weaker. In contrast, HPN, GCNT1 and CSRP1 display bidirectional regulatory characteristics. Most other genes cluster around zero, indicating a relatively minor impact on the model. (**Figure 3G**). The waterfall plot further demonstrated that SLC14A1 (node weight 7.23, Δ = -0.287) is the primary negative regulatory hub, causing the predicted model output (f(x) = 0.098) to be significantly lower than the baseline expectation (E[f(x)] = 0.616) (**Figure 3H**). Ultimately, the clustered heatmap displays the calculated SHAP values for these genes across the entire set of samples (**Figure 3I**).

### 3.4 Expression profiles and prognostic relevance of candidate genes in prostate cancer

**Figure 4:**
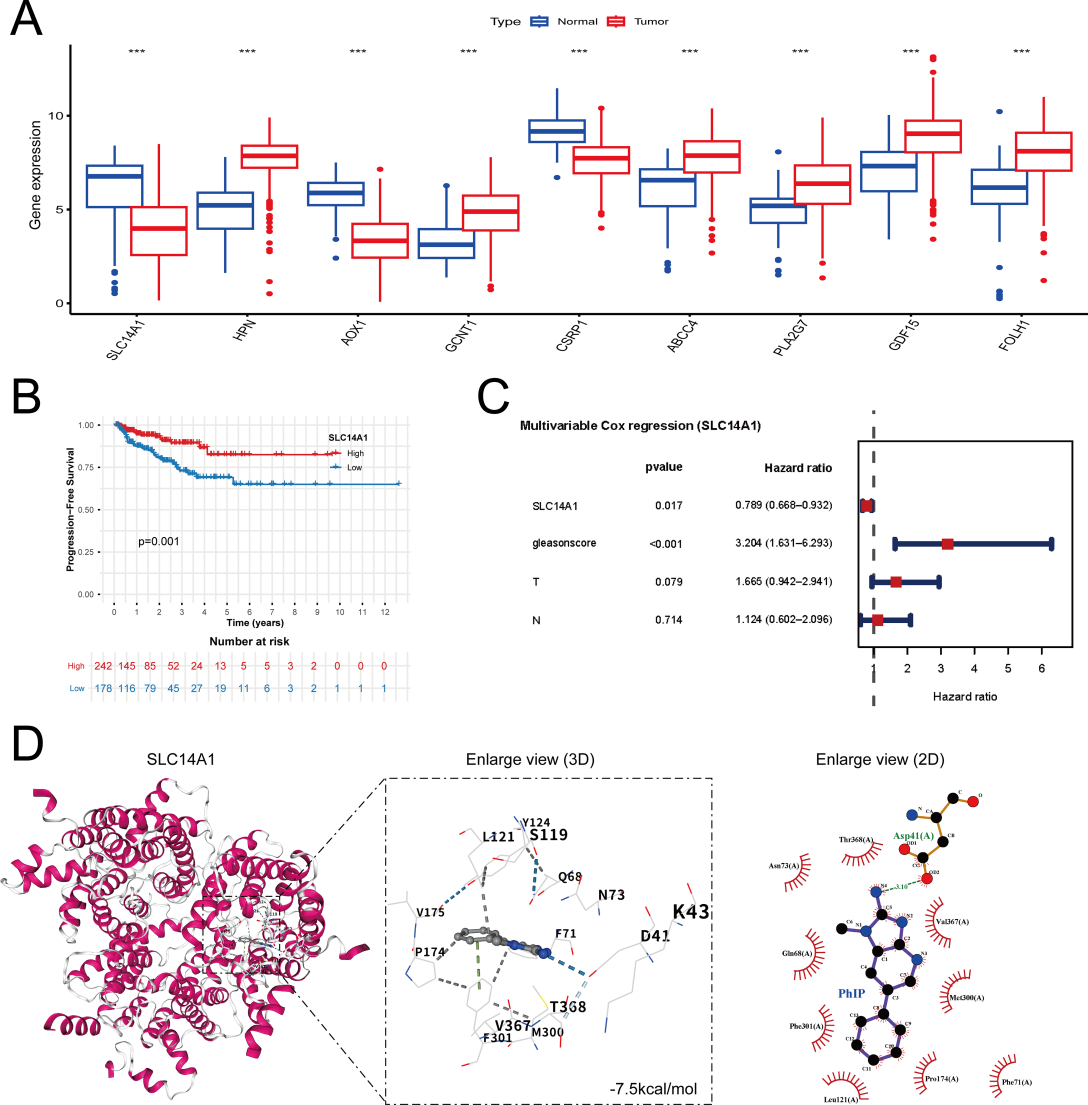
Expression pattern, prognostic significance, and molecular docking analysis of SLC14A1 in prostate cancer. **(A)** Boxplots showing the differential expression of nine candidate genes between prostate cancer (PCa) tissues and adjacent normal tissues. Each boxplot represents one gene. Differences between groups were evaluated using the Wilcoxon rank-sum test. **(B)** Kaplan–Meier analysis of progression-free survival (PFS) stratified by SLC14A1 expression level. Patients were divided into high- and low-expression groups based on the median gene expression value. Statistical significance was assessed using the log-rank test, and the number of patients at risk is shown below the survival curve. **(C)** Forest plot of multivariate Cox regression analysis evaluating the prognostic significance of SLC14A1 and other clinicopathological variables in prostate cancer. Hazard ratios (HR) and corresponding 95% confidence intervals (CI) are presented. **(D)** Molecular docking analysis between PhIP and SLC14A1. The left panel shows the three-dimensional structure of the SLC14A1 protein. The middle panel displays the docking conformation of PhIP within the predicted binding pocket. The right panel presents a two-dimensional interaction diagram highlighting hydrogen bonds and hydrophobic interactions between PhIP and surrounding residues. *** indicates p < 0.001.

An examination of nine candidate genes, including SLC14A1, AOX1, CSRP1, HPN, and GCNT1, demonstrated significant differential expression between tumor tissues and adjacent normal tissues (**Figure 4A**). Notably, SLC14A1 and AOX1 exhibited pronounced downregulation in tumor specimens, whereas HPN and several other genes were upregulated. Kaplan–Meier survival analyses revealed that elevated expression levels of SLC14A1, AOX1, CSRP1, and GCNT1 were significantly correlated with extended biochemical recurrence (BCR)-based progression-free survival (PFS). Following false discovery rate (FDR) correction for multiple comparisons, SLC14A1 and CSRP1 maintained statistical significance in the Kaplan–Meier analysis. However, multivariable Cox regression analysis identified only SLC14A1 expression, as a key gene, as an independent prognostic factor (**Figures 4B, C**; **Supplementary Figure 1**). Collectively, these findings suggest that the differentially expressed genes, particularly SLC14A1, are strongly associated with BCR in prostate cancer. Nonetheless, the independent prognostic utility of these genes warrants further validation through studies involving larger patient cohorts and prospective designs.

### 3.5 Molecular docking validation

To systematically assess the potential binding affinity between PhIP and the core gene SLC14A1, as identified via machine learning techniques, a high-precision molecular docking methodology was utilized to investigate their structure–function relationship. The findings demonstrated that PhIP could form a stable complex with SLC14A1, exhibiting a binding free energy (ΔG_bind) below −5.0 kcal·mol^-^¹ (**Table 1**). Visualization of the three-dimensional binding conformation alongside the two-dimensional interaction map (**Figure 4D**) further elucidated that PhIP participates in a conserved network of hydrophobic interactions and hydrogen bonds at either the active or allosteric sites of SLC14A1, thereby stabilizing the docking conformation. Moreover, three-dimensional binding conformations and two-dimensional interaction maps were generated for PhIP in complex with four additional core genes ranked highly by SHAP analysis (**Supplementary Figure 2**). The binding free energies (ΔG_bind) for these genes were also found to be below −5.0 kcal·mol^-^¹ (**Table 1**). Taken together, these results provide structural evidence supporting a potential direct molecular interaction between PhIP and the core gene SLC14A1. This suggests that SLC14A1 may be involved in PhIP-associated prostate cancer (PhIP-PCa) via a ligand–protein interaction mechanism.

**Table 1:** Binding energy between core genes and PhIP.

Protein	PDB ID or UniProt ID	Ligand	Binding energy (kcal/mol)
SLC14A1	Q13336	PhIP	-7.5
HPN	P05981		-5.4
GCNT1	Q02742		-7.0
AOX1	Q06278		-7.8
CSRP1	P21291		-7.2

### 3.6 Molecular dynamics simulation of SLC14A1–PhIP interaction

**Figure 5:**
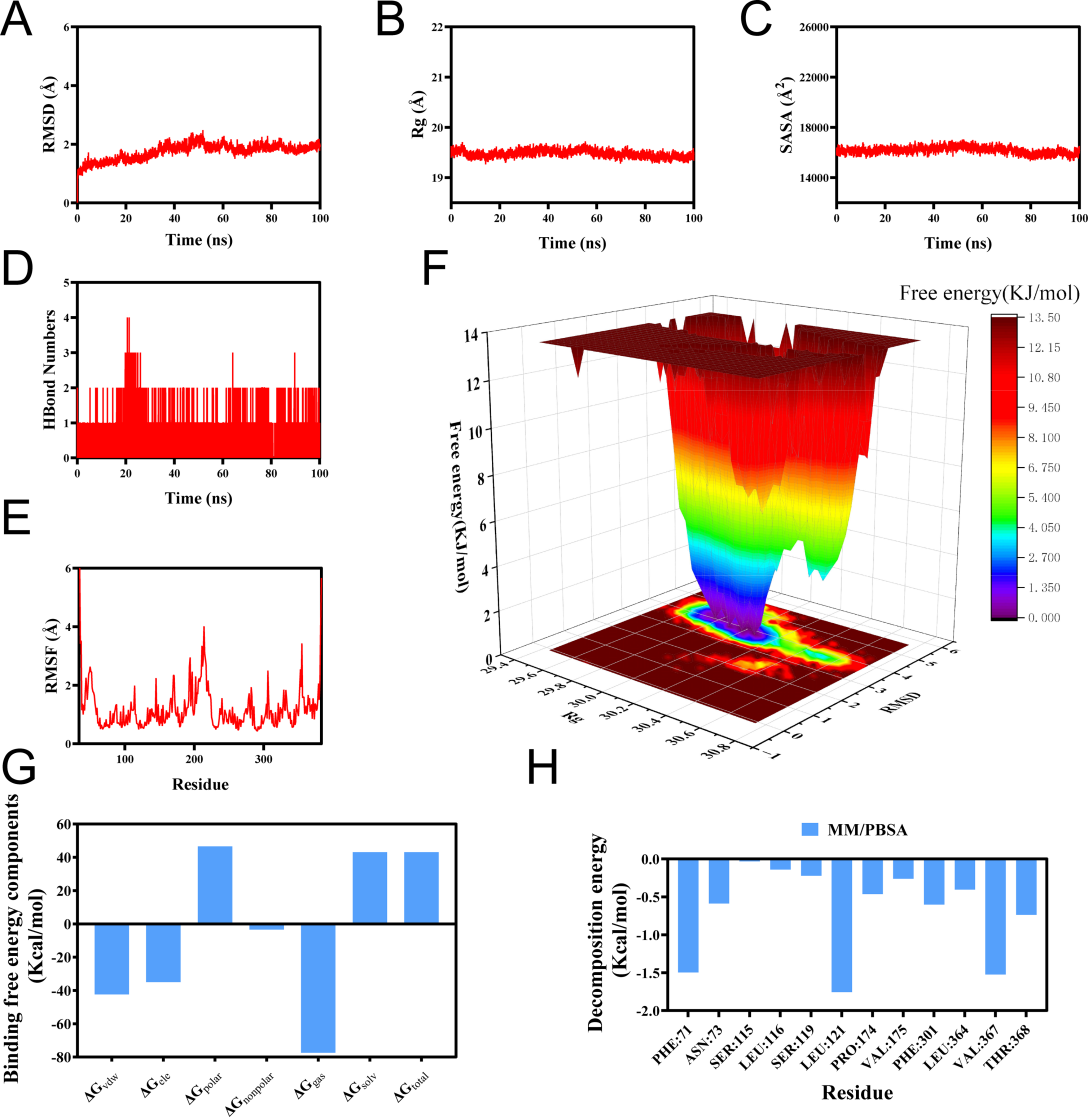
Quantitative analysis of the 100-ns molecular dynamics trajectory for the SLC14A1–PhIP complex. **(A)** Root-mean-square deviation (RMSD). The Cα RMSD plateaued after approximately 60 ns and remained at 1.9 ± 0.2 Å for the remainder of the simulation, indicating that the system reached thermodynamic equilibrium within 100 ns and that ligand binding does not induce appreciable conformational drift. **(B)** Radius of gyration (Rg). The Rg value remained stable at 19.4 ± 0.3 Å, with a fluctuation amplitude <1.5%, revealing no expansion or compression of the protein scaffold and confirming the structural compactness of the complex throughout the trajectory. **(C)** Solvent-accessible surface area (SASA). SASA exhibited only minor fluctuations and no systematic trend, indicating that PhIP association causes negligible changes to the exposed protein surface and that the overall architecture of the complex remains preserved. **(D)** Hydrogen bonding. The number of intermolecular hydrogen bonds fluctuated between 0 and 4, with an average of approximately 2, highlighting the formation of a persistent hydrogen-bond network that contributes to the stability of the SLC14A1–PhIP complex. **(E)** Root-mean-square fluctuation (RMSF). The mean RMSF per residue was 1.9 Å, with 90% of residues exhibiting RMSF values <3 **(Å)** The binding-pocket region showed maximal fluctuations of 2.1 Å, reflecting low flexibility and high structural rigidity. **(F)** Free-energy landscape (FEL). The FEL constructed using RMSD and Rg, revealed a single, narrow low-energy basin (blue region), signifying that the complex predominantly occupies a stable conformational state without any discernible metastable basins. **(G)** MM/PBSA binding free energy. The calculated binding free energy, ΔG_bind = −19.79 kcal·mol^-^¹, is strongly negative, evidencing high binding affinity and corroborating the experimentally observed activity. **(H)** Per-residue energy decomposition. Leu121, Val367, Phe71, Thr368, Asn73, and Phe301 were identified as the primary energetic contributors, suggesting their pivotal roles in ligand recognition and complex stabilization.

We further analyzed the molecular docking results using MD simulations. Taking the complex formed by SLC14A1 and PhIP as a representative example, the 100 ns MD trajectory showed that the backbone RMSD of the SLC14A1-PhIP complex stabilized at 1.9 ± 0.2 Å after 60 ns (**Figure 5A**). The Rg and SASA stabilized at 19.4 ± 0.3 Å and 16,100 ± 200 Å², respectively, with no significant expansion or contraction observed (**Figures 5B, C**). The average number of hydrogen bonds was 2.1 ± 0.8 (**Figure 5D**), and RMSF values below 3 Å indicated notable rigidity at the interface (**Figure 5E**). The free energy landscape based on RMSD and Rg displayed a single deep blue low-energy basin, occupying more than 85% of the sampling time (**Figure 5F**). MM/PBSA calculations yielded a binding free energy (ΔG_bind) of –19.8 ± 1.3 kcal/mol, with energy decomposition revealing that Leu121, Val367, Phe71, Thr368, Asn73, and Phe301 contributed over 70% of the interaction energy (**Figures 5G, H**). Taken together, these results indicate that the small molecule forms a high-affinity, low-flexibility, and thermodynamically stable complex with the target protein through the synergistic effects of hydrogen bonding and a hydrophobic core. This finding further supports its potential mechanism of inducing prostate cancer via a “ligand-protein” interaction model.

### 3.7 Validation of PhIP-induced cytotoxicity in RWPE-1 cells and SLC14A1 downregulation in PC-3 cells

**Figure 6:**
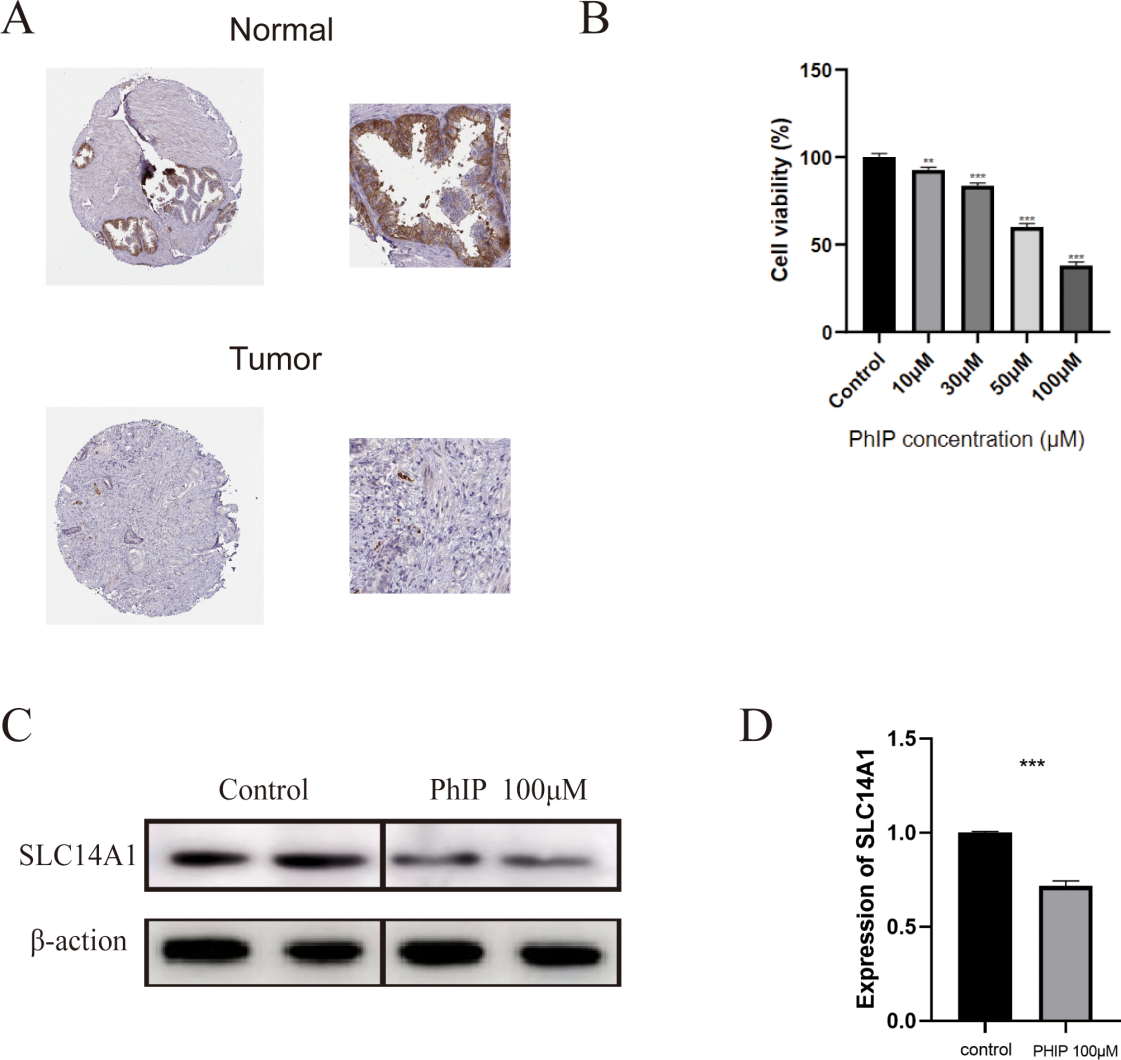
PhIP exposure induces cytotoxicity in RWPE-1 cells and downregulates SLC14A1 expression in PC-3 cells. **(A)** Immunohistochemical staining of SLC14A1 in prostate cancer tissues and normal prostate tissues based on data from the HPA database. **(B)** Cell viability of RWPE-1 cells after treatment with increasing concentrations of PhIP for 48 h, as determined by CCK-8 assay. **(C)** Protein expression levels of SLC14A1 in PC-3 cells following PhIP treatment, as assessed by Western blot. **(D)** Relative mRNA expression levels of SLC14A1 in PC-3 cells after PhIP treatment, as determined by qRT-PCR (**p < 0.01, ***p < 0.001).

Building upon the findings from molecular docking and molecular dynamics simulations, we conducted experimental investigations to evaluate the cytotoxic effects of PhIP and its influence on SLC14A1 expression in prostate epithelial cells. Immunohistochemical analysis utilizing data from the Human Protein Atlas (HPA) database indicated that SLC14A1 protein levels were significantly diminished in tumor tissues, as evidenced by weak staining intensity and a low percentage of positively stained cells. In contrast, normal tissues exhibited stronger cytoplasmic staining intensity and a higher proportion of positive cells (**Figure 6A**). To directly assess the impact of PhIP, RWPE-1 cells were exposed to escalating concentrations of the compound. Cell viability assays revealed a dose-dependent cytotoxic response (**Figure 6B**). Subsequent analyses involving protein quantification and quantitative reverse transcription PCR (qRT-PCR) demonstrated that PhIP treatment led to a marked downregulation of SLC14A1 expression in prostate cancer cells (**Figures 6C, D**). Collectively, these experimental results substantiate that PhIP induces cytotoxicity in human prostatic epithelial cells and concomitantly suppresses SLC14A1 expression.

### 3.8 Regulatory function of SLC14A1 in modulating tumor microenvironment infiltration patterns in prostate cancer

**Figure 7:**
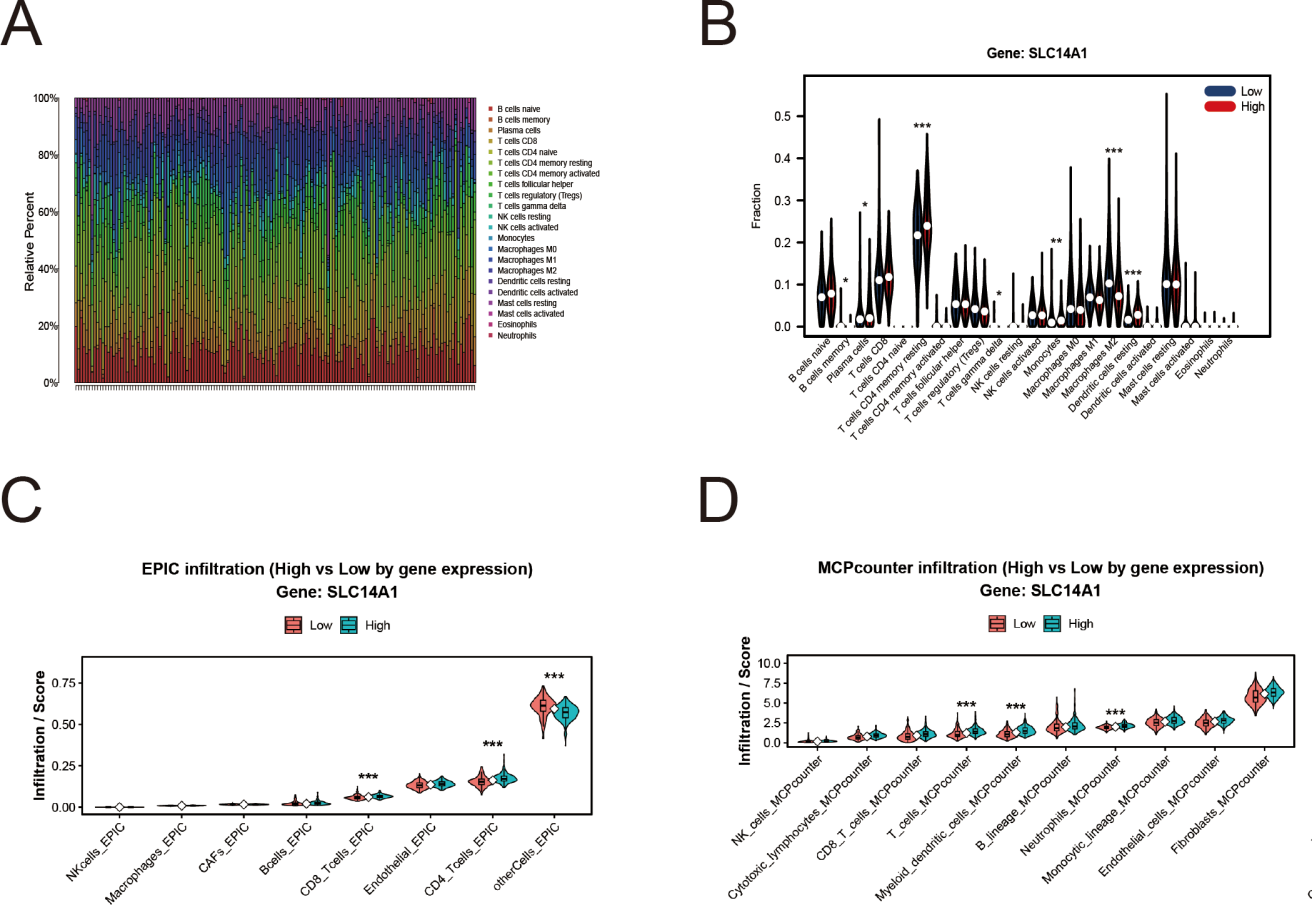
Association between SLC14A1 expression and immune cell infiltration in the tumor microenvironment. **(A)**Stacked bar plot illustrating the relative proportions of 22 tumor-infiltrating immune cell types in prostate cancer samples, as estimated by the CIBERSORT algorithm. Each bar represents an individual sample, and different colors correspond to distinct immune cell subsets. **(B)** Violin plots comparing immune cell fractions between high- and low-SLC14A1 expression groups based on CIBERSORT results.Statistical significance is indicated by asterisks (*p < 0.05, **p < 0.01, ***p < 0.001). **(C)** Immune cell infiltration levels estimated by the EPIC algorithm, comparing high- and low-SLC14A1 expression groups. **(D)** Immune cell infiltration analysis using the MCPcounter method, comparing high- and low-SLC14A1 expression groups.

The tumor microenvironment (TME) plays a pivotal role in the progression of prostate cancer. Initial observations revealed substantial heterogeneity in immune cell infiltration among prostate cancer samples (**Figure 7A**). To further characterize the association between SLC14A1 expression and the immune landscape, the relative abundance of tumor-infiltrating immune cells was estimated using the CIBERSORT algorithm. Correlation analysis demonstrated that SLC14A1 expression was significantly associated with multiple immune cell populations, particularly T cell subsets and macrophage-related signatures. These relationships were visualized as a hierarchically clustered heatmap (**Figure 7B**).

To enhance robustness, immune infiltration was additionally evaluated using the EPIC and MCPcounter algorithms (**Figures 7C, D**). Consistent trends were observed across these methods, although variations in effect size were noted, reflecting methodological differences in immune cell estimation. Collectively, these results suggest that SLC14A1 expression is associated with the heterogeneity of the intratumoral immune microenvironment in prostate cancer.

### 3.9 Single-cell RNA-seq analysis of SLC14A1 expression

**Figure 8:**
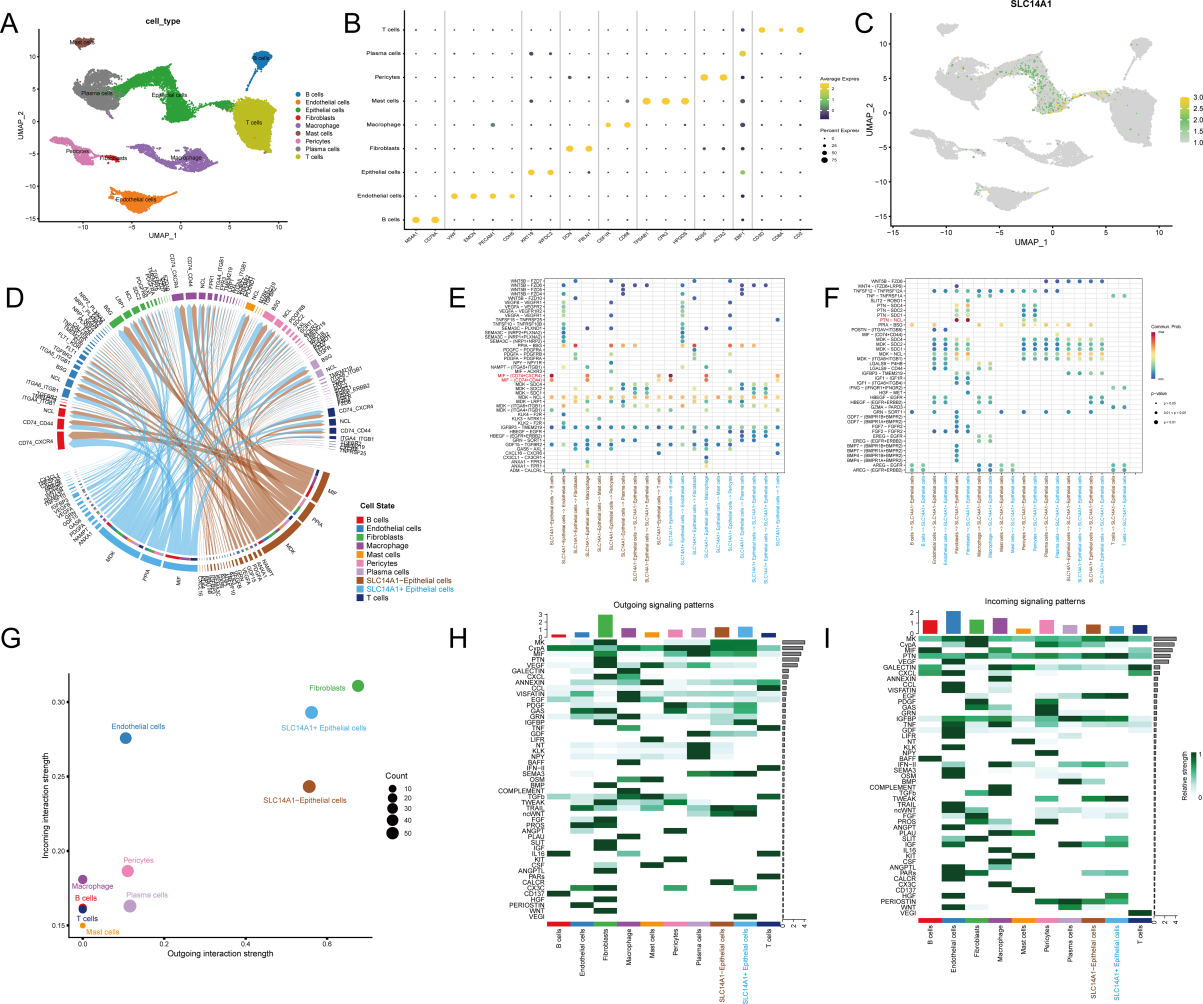
Analysis of single-cell RNA sequencing data in the prostate cancer microenvironment. **(A)** UMAP visualization of major cell populations identified from the single-cell transcriptome dataset, including B cells, endothelial cells, epithelial cells, fibroblasts, macrophages, mast cells, pericytes, plasma cells, and T cells.**(B)** Bubble plot showing the expression proportion (dot size) and average expression level (color gradient) of marker genes across different cell types, supporting the accuracy of cell type annotation.**(C)** UMAP feature plots displaying the expression distribution of representative genes SLC14A1, HPN, GCNT1, AOX1, and CSRP1 in the single-cell dataset.**(D)** Chord diagram illustrating cell–cell communication networks mediated by ligand–receptor interactions. SLC14A1^-^ epithelial cells and SLC14A1^+^ epithelial cells exhibit extensive interactions with multiple tumor microenvironment cell types.**(E)** Bubble plot showing outgoing signaling patterns mediated by ligand–receptor interactions between different cell populations.**(F)** Bubble plot showing incoming signaling patterns mediated by ligand–receptor interactions between different cell populations.**(G)** Scatter plot of cell communication centrality comparing outgoing (x-axis) and incoming (y-axis) interaction strength across cell types.**(H)** Heatmap of outgoing signaling pathway communication patterns among different cell populations.**(I)** Heatmap of incoming signaling pathway communication patterns among different cell populations.

To further investigate the cellular localization and expression characteristics of SLC14A1 in prostate cancer tissues, we performed an integrated analysis of the GSE193337 single-cell transcriptome dataset. We successfully annotated and identified nine major cell populations, including B cells, endothelial cells, epithelial cells, fibroblasts, macrophages, mast cells, pericytes, plasma cells, and T cells (**Figure 8A**). The feature gene expression bubble plot validated the specificity and biological accuracy of these subgroup annotations (**Figure 8B**). Notably, single-cell localization analysis showed that SLC14A1 was significantly enriched in epithelial cells in prostate cancer (**Figure 8C**).

To explore the potential mechanisms linking SLC14A1 expression to PCa progression, we conducted a cell–cell communication analysis. The results revealed extensive crosstalk between these two epithelial cell types and other cellular components within TME (**Figure 8D**). Further analysis of ligand–receptor (L-R) pairs indicated that both SLC14A1-positive (SLC14A1^+^) and SLC14A1-negative (SLC14A1^-^) epithelial cells exhibited relatively strong macrophage migration inhibitory factor (MIF) secretion signals and pleiotrophin (PTN) reception signals (**Figures 8E, F**). Notably, compared with SLC14A1^-^ epithelial cells, SLC14A1^+^ epithelial cells displayed comparable outgoing signal strength but significantly higher incoming signal intensity, suggesting a more active role as signal receivers within the TME (**Figure 8G**). Signaling pathway heatmap analysis further revealed distinct communication patterns between the two cell subsets. Regarding outgoing signals (**Figure 8H**), although overall outgoing strength was similar, notable differences were observed in specific pathways. SLC14A1^+^ epithelial cells exhibited stronger outgoing signals enriched in IGFBP (insulin-like growth factor binding protein), SEMA3 (semaphorin 3, an angiogenesis inhibitor), ncWNT (non-canonical WNT), CX3C (chemokine signaling), and VEGI (vascular endothelial growth inhibitor), most of which are associated with tumor suppression. In contrast, SLC14A1^-^ epithelial cells showed enhanced outgoing activity in pathways including TRAIL (TNF-related apoptosis-inducing ligand) and CALCR (calcitonin receptor signaling, linked to bone metastasis). For incoming signals (**Figure 8I**), SLC14A1^+^ epithelial cells exhibited significantly stronger reception of MK (Midkine), PTN (pleiotrophin), EGF (epidermal growth factor), and TWEAK signaling. These observations may be attributable to SLC14A1-mediated blockade of downstream signal transduction. Collectively, the loss of SLC14A1 expression may alter the sensitivity of prostate cancer epithelial cells to microenvironmental signals, thereby facilitating tumor progression.

## 4 Discussion

In this study, multi-omics data were integrated with machine learning and bioinformatics methods to systematically analyze the potential molecular association between PhIP exposure and the pathogenesis of prostate cancer. A total of 17 candidate genes were identified through combined network toxicology and multi-omics analysis, of which 9 core genes (SLC14A1, HPN, AOX1, GCNT1, CSRP1, ABCC4, PLA2G7, GDF15, and FOLH1) demonstrated significant importance. Differential expression analysis revealed that SLC14A1, AOX1, and CSRP1 were significantly downregulated, whereas HPN, GCNT1, ABCC4, PLA2G7, GDF15, and FOLH1 were significantly upregulated in prostate cancer tissues. Validation using machine learning models showed that these 9 gene features exhibited excellent diagnostic efficiency. SHAP interpretability analysis identified the top 5 genes as the primary contributors, with SLC14A1 (SHAP = 0.121) being the most influential predictive variable. Immune and single-cell localization analyses suggest that PhIP may affect tumor progression by modulating the immune microenvironment in prostate cancer. The results of molecular docking and molecular dynamics simulations support the biological plausibility of these findings, demonstrating that PhIP forms a high-affinity, low-flexibility, and thermodynamically stable complex with specific amino acid residues of SLC14A1 through the synergistic effects of hydrogen bonding and a hydrophobic core (ΔG_bind = -7.5 kcal/mol). Further experiments confirmed that PhIP exposure significantly affects the activity, morphology, and expression levels of key target proteins in prostate cells. In summary, PhIP may promote the initiation and progression of prostate cancer by disrupting the normal functions of these proteins, particularly SLC14A1, potentially through direct molecular interactions.

The type B urine transporter, a member of the urinary transporter family, plays a particularly important role in regulating urine concentration (31). Its encoding gene, Solute Carrier Family 14 Member 1 (SLC14A1), also known as UTB, is located on chromosome 18q12.3, adjacent to the SLC14A2 locus (32). SLC14A1 is widely expressed in various tissues, including red blood cells, renal parenchyma, and bladder epithelium. The passive transport of urea mediated by SLC14A1 not only contributes to the establishment of the osmotic gradient in the inner layer of the renal medulla but also protects extrarenal cells from hyperosmolar toxicity by reducing intracellular urea concentration (33). Further studies have proved that the SLC14A1 gene is a novel inhibitor of urothelial carcinoma, exerting significant suppressive effects on cell viability, proliferation, migration, invasion, tumor growth, and metastasis (34). In PCa, multi-omics integration and functional experiments confirmed that the expression of SLC14A1 is significantly downregulated in prostate cancer tissues. Moreover, its low expression level is independently associated with poor prognosis. Mechanistically, SLC14A1 inhibits the CDK1/CCNB1 and mTOR/MMP-9 signaling pathways, thereby blocking the cell cycle and reducing invasive capacity. Additionally, the gene is enriched in the basal cell subset of the prostate, suggesting that SLC14A1 may serve as an epigenetic marker and a potential therapeutic target for prostate cancer progression (35). This is consistent with our research findings. SLC14A1, identified as the most important negative regulatory factor, can interact with PhIP to produce effects. The impact of PhIP on prostate cancer may result from its direct binding, which influences the expression of key proteins, thereby affecting the cell cycle and the immune microenvironment.

This study has several limitations. First, although the current sample size provides sufficient exploratory statistical power, it may restrict the generalizability of the findings and limit their applicability to a broader population. Second, while some confounding factors such as age, genetic background, and lifestyle were partially controlled, residual confounding may still affect the accuracy of effect estimation. Third, the biological relevance and predictive accuracy of the model require validation through systematic *in vitro* and *in vivo* experiments. Fourth, although our *in vitro* experiments demonstrated reduced viability and gene expression changes after PhIP exposure, we did not include relevant confirmatory experiments to distinguish general cytotoxicity from carcinogen-associated injury. Fifth, this study did not perform direct experimental validation of the effects of PhIP on immune cells, and therefore the associated immune regulatory mechanisms require further confirmation in future research. The mechanistic interpretation should be considered preliminary and warrants further validation. Future studies should: (1) expand the cohort size and enhance sample diversity, prioritizing populations exposed to PhIP to improve risk stratification; (2) integrate multi-omics approaches to comprehensively elucidate the mechanisms of PhIP toxicity and carcinogenesis; (3) develop chemoprevention strategies centered on SLC14A1; and (4) analyze the dynamic regulatory networks of epigenetics involved in PhIP-induced carcinogenesis. Implementing these strategies will substantially enhance the scientific rigor and public health relevance of the research findings.

## Data Availability

The original contributions presented in the study are included in the article/**Supplementary Material**. Further inquiries can be directed to the corresponding authors.
