# Supplementary material for: PhIP-driven prostate cancer involves key molecular regulators and immune microenvironment modulation
Source: Front Immunol. 2026 Apr 10;17:1782240. doi: 10.3389/fimmu.2026.1782240 (PMC13106171; doi:10.3389/fimmu.2026.1782240)
Supplement: Supplementary file 1 [file DataSheet1.docx]

Supplementary Material

Supplementary Figure 1. Kaplan–Meier survival analysis and multivariable Cox regression of the remaining four candidate genes.


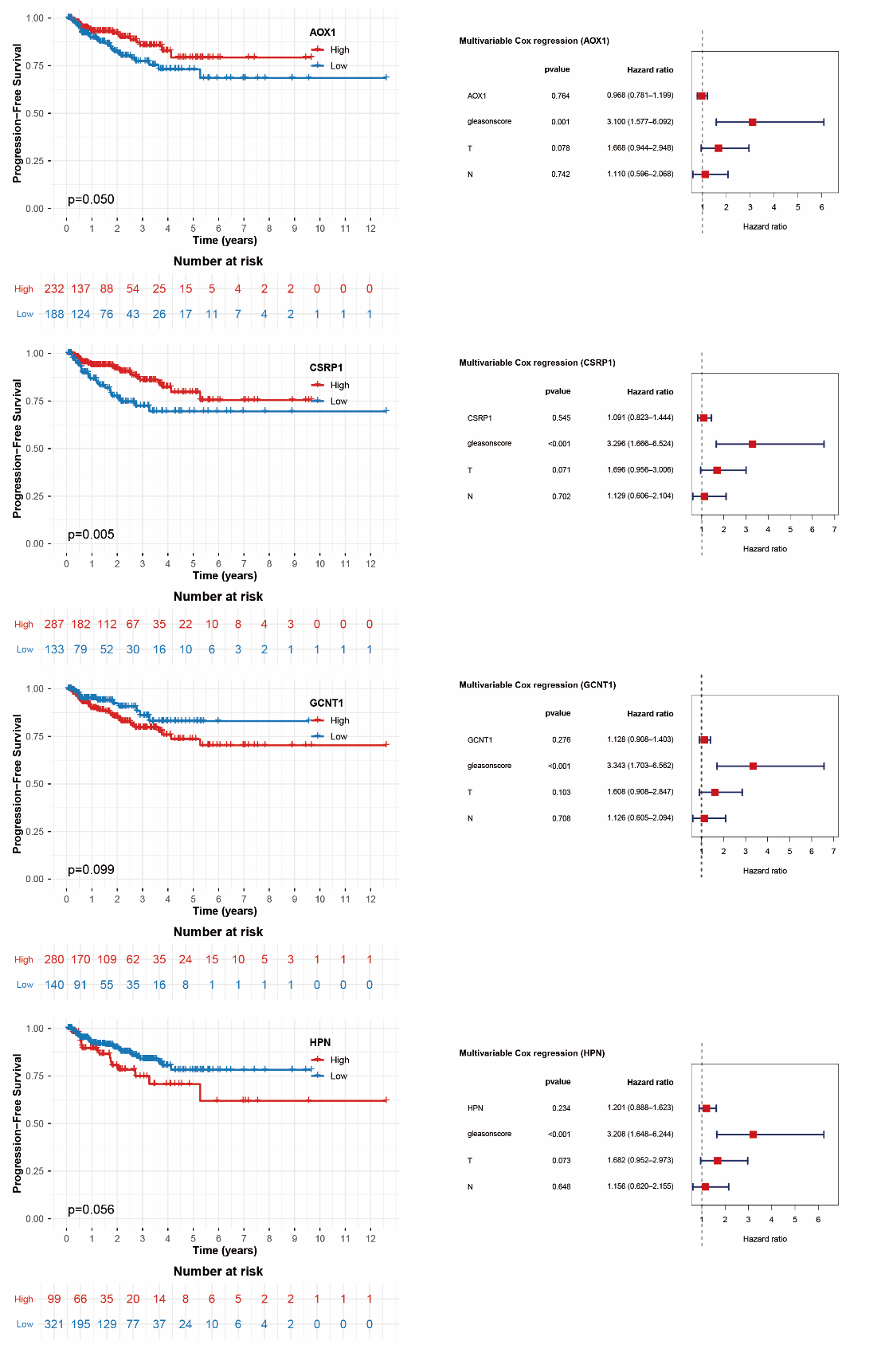


**Supplementary Figure 2.** Molecular docking analysis of PhIP with four additional candidate genes (GCNT1, HPN, CSRP1, and AOX1).


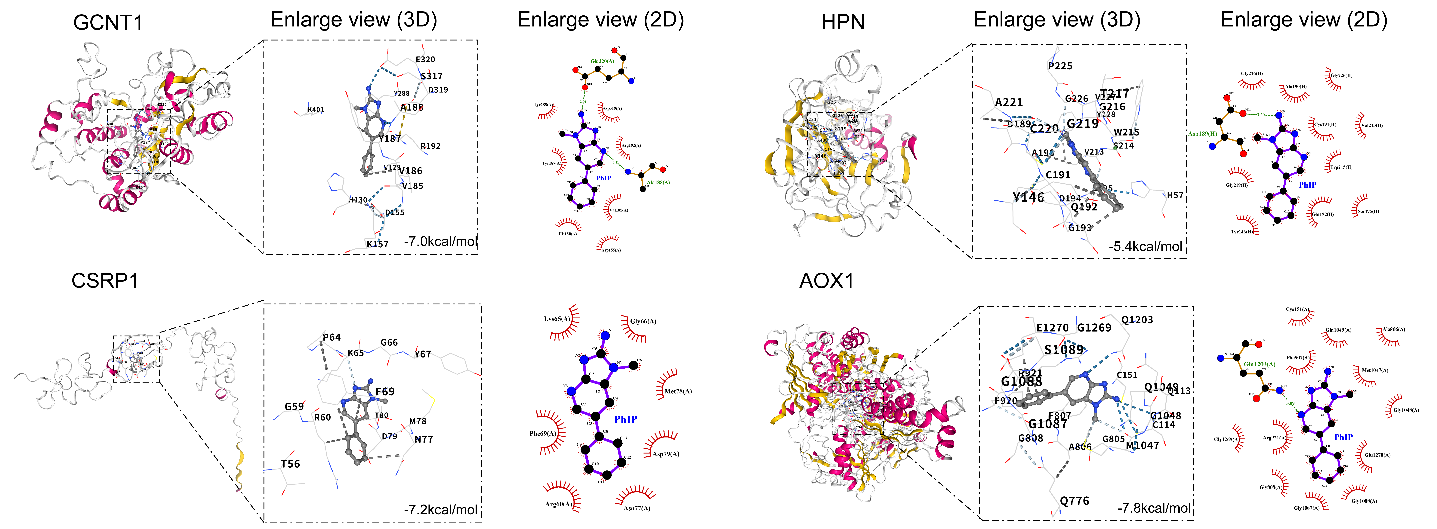


**Supplementary Figure 3.** Western blot image of β-actin used as the loading control.


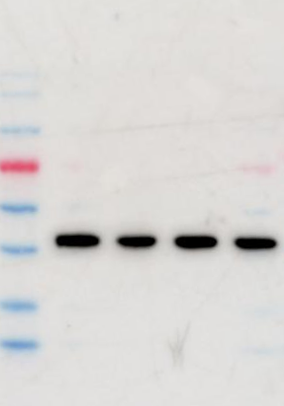


**Supplementary Figure 4** Western blot band image of SLC14A1.


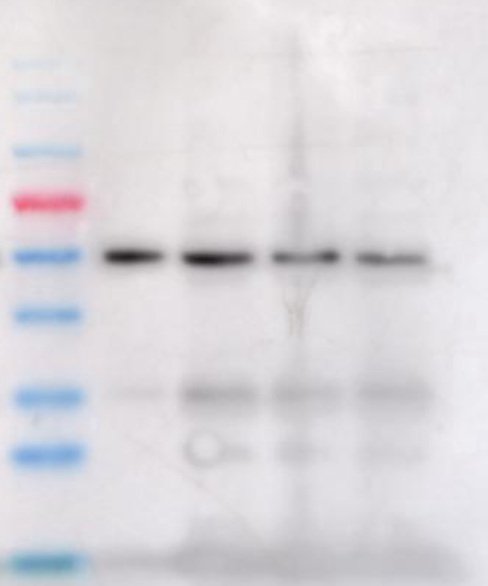


**Supplementary Table1**. Comprehensive keyword list for filtering cancer-related pathways

| **Category** | **Keyword** |
| --- | --- |
| General Cancer Terms | CANCER |
| General Cancer Terms | CARCINOMA |
| General Cancer Terms | ONCOGENIC |
| General Cancer Terms | TUMOR |
| General Cancer Terms | MALIGNANT |
| General Cancer Terms | NEOPLASM |
| Cell Cycle and Apoptosis | P53 |
| Cell Cycle and Apoptosis | CELL_CYCLE |
| Cell Cycle and Apoptosis | APOPTOSIS |
| Cell Cycle and Apoptosis | CHECKPOINT |
| DNA Damage and Repair | DNA_REPAIR |
| DNA Damage and Repair | MISMATCH_REPAIR |
| DNA Damage and Repair | HOMOLOGOUS_RECOMBINATION |
| DNA Damage and Repair | NUCLEOTIDE_EXCISION_REPAIR |
| DNA Damage and Repair | BASE_EXCISION_REPAIR |
| DNA Damage and Repair | DOUBLE_STRAND_BREAK |
| DNA Damage and Repair | DNA_ADDUCT |
| Canonical Oncogenic Signaling Pathways | PI3K |
| Canonical Oncogenic Signaling Pathways | AKT |
| Canonical Oncogenic Signaling Pathways | MTOR |
| Canonical Oncogenic Signaling Pathways | MAPK |
| Canonical Oncogenic Signaling Pathways | ERK |
| Canonical Oncogenic Signaling Pathways | JAK_STAT |
| Canonical Oncogenic Signaling Pathways | WNT |
| Canonical Oncogenic Signaling Pathways | NOTCH |
| Canonical Oncogenic Signaling Pathways | HEDGEHOG |
| Canonical Oncogenic Signaling Pathways | TGF_BETA |
| Canonical Oncogenic Signaling Pathways | TGF_B |
| Canonical Oncogenic Signaling Pathways | NF_KAPPA_B |
| Canonical Oncogenic Signaling Pathways | NF_KB |
| Tumor Progression & Microenvironment | HYPOXIA |
| Tumor Progression & Microenvironment | ANGIOGENESIS |
| Tumor Progression & Microenvironment | METASTASIS |
| Tumor Progression & Microenvironment | EMT |
| Xenobiotic Metabolism and Chemical Carcinogenesis | XENOBIOTICS |
| Xenobiotic Metabolism and Chemical Carcinogenesis | METABOLISM_OF_XENOBIOTICS |
| Xenobiotic Metabolism and Chemical Carcinogenesis | CYTOCHROME_P450 |
| Xenobiotic Metabolism and Chemical Carcinogenesis | CYP450 |
| Xenobiotic Metabolism and Chemical Carcinogenesis | GLUTATHIONE |
| Xenobiotic Metabolism and Chemical Carcinogenesis | AHR |
| Xenobiotic Metabolism and Chemical Carcinogenesis | ARNT |
| Xenobiotic Metabolism and Chemical Carcinogenesis | NRF2 |
| Xenobiotic Metabolism and Chemical Carcinogenesis | KEAP1 |
| Xenobiotic Metabolism and Chemical Carcinogenesis | SENOBIOTIC |
| Oxidative Stress Response | OXIDATIVE_STRESS |
| Oxidative Stress Response | REACTIVE_OXYGEN |
| Prostate Cancer–Specific Terms | PROSTATE |
| Prostate Cancer–Specific Terms | ANDROGEN |
| Prostate Cancer–Specific Terms | AR |
| Prostate Cancer–Specific Terms | PSA |
| Prostate Cancer–Specific Terms | TMPRSS2 |
